# The Metabochip, a Custom Genotyping Array for Genetic Studies of Metabolic, Cardiovascular, and Anthropometric Traits

**DOI:** 10.1371/journal.pgen.1002793

**Published:** 2012-08-02

**Authors:** Benjamin F. Voight, Hyun Min Kang, Jun Ding, Cameron D. Palmer, Carlo Sidore, Peter S. Chines, Noël P. Burtt, Christian Fuchsberger, Yanming Li, Jeanette Erdmann, Timothy M. Frayling, Iris M. Heid, Anne U. Jackson, Toby Johnson, Tuomas O. Kilpeläinen, Cecilia M. Lindgren, Andrew P. Morris, Inga Prokopenko, Joshua C. Randall, Richa Saxena, Nicole Soranzo, Elizabeth K. Speliotes, Tanya M. Teslovich, Eleanor Wheeler, Jared Maguire, Melissa Parkin, Simon Potter, N. William Rayner, Neil Robertson, Kathleen Stirrups, Wendy Winckler, Serena Sanna, Antonella Mulas, Ramaiah Nagaraja, Francesco Cucca, Inês Barroso, Panos Deloukas, Ruth J. F. Loos, Sekar Kathiresan, Patricia B. Munroe, Christopher Newton-Cheh, Arne Pfeufer, Nilesh J. Samani, Heribert Schunkert, Joel N. Hirschhorn, David Altshuler, Mark I. McCarthy, Gonçalo R. Abecasis, Michael Boehnke

**Affiliations:** 1 Medical Population Genetics, The Broad Institute of Harvard and Massachusetts Institute of Technology, Cambridge, Massachusetts, United States of America; 2 Department of Pharmacology, University of Pennsylvania Perelman School of Medicine, Philadelphia, Pennsylvania, United States of America; 3 Department of Biostatistics, Center for Statistical Genetics, University of Michigan, Ann Arbor, Michigan, United States of America; 4 Laboratory of Genetics, National Institute on Aging, National Institutes of Health, Baltimore, Maryland, United States of America; 5 Divisions of Endocrinology and Genetics and Program in Genomics, Children's Hospital, Boston, Massachusetts, United States of America; 6 Istituto di Ricerca Genetica e Biomedica, Consiglio Nazionale delle Ricerche (CNR), Monserrato, Italy; 7 Dipartimento di Scienze Biomediche, Università di Sassari, Sassari, Italy; 8 Genome Technology Branch, National Human Genome Research Institute, Bethesda, Maryland, United States of America; 9 Universität zu Lübeck, Medizinische Klinik II, and Nordic Center of Cardiovascular Research, Lübeck, Germany; 10 Genetics of Complex Traits, Peninsula College of Medicine and Dentistry, University of Exeter, Exeter, United Kingdom; 11 Department of Epidemiology and Preventive Medicine, University Hospital Regensburg, Regensburg, Germany; 12 Helmholtz Zentrum München—German Research Center for Environmental Health, Institute of Epidemiology, Neuherberg, Germany; 13 Clinical Pharmacology and Barts and the London Genome Centre, William Harvey Research Institute, Barts and the London School of Medicine, Queen Mary University of London, London, United Kingdom; 14 MRC Epidemiology Unit, Institute of Metabolic Science, Addenbrooke's Hospital, Cambridge, United Kingdom; 15 Wellcome Trust Centre for Human Genetics, University of Oxford, Oxford, United Kingdom; 16 Oxford Centre for Diabetes, Endocrinology, and Metabolism, Churchill Hospital, University of Oxford, Oxford, United Kingdom; 17 Center for Human Genetic Research, Massachusetts General Hospital, Boston, Massachusetts, United States of America; 18 Department of Anesthesia, Critical Care and Pain Medicine, Massachusetts General Hospital, Boston, Massachusetts, United States of America; 19 Wellcome Trust Sanger Institute, Hinxton, Cambridge, United Kingdom; 20 Department of Internal Medicine, Division of Gastroenterology and Center for Computational Medicine and Bioinformatics, University of Michigan, Ann Arbor, Michigan, United States of America; 21 University of Cambridge Metabolic Research Laboratories, Institute of Metabolic Science, Addenbrooke's Hospital, Cambridge, United Kingdom; 22 Cardiovascular Research Center and Cardiology Division, Massachusetts General Hospital, Boston, Massachusetts, United States of America; 23 Department of Medicine, Harvard Medical School, Boston, Massachusetts, United States of America; 24 Institute of Human Genetics, Klinikum Rechts der Isar Technische Universität München, Munich, Germany; 25 Institute of Human Genetics, Helmholtz Zentrum München, Deutsches Forschungszentrum für Gesundheit und Umwelt, Neuherberg, Germany; 26 EURAC Center of Biomedicine, Bolzano, Italy; 27 Department of Cardiovascular Sciences, Glenfield Hospital, University of Leicester, Leicester, United Kingdom; 28 Leicester NIHR Biomedical Research Unit in Coronary Artery Disease, Glenfield Hospital, Leicester, United Kingdom; 29 Department of Genetics, Harvard Medical School, Boston, Massachusetts, United States of America; 30 Department of Molecular Biology, Harvard Medical School, Boston, Massachusetts, United States of America; 31 Diabetes Unit, Massachusetts General Hospital, Boston, Massachusetts, United States of America; 32 Oxford NIHR Biomedical Research Centre, Churchill Hospital, Oxford, United Kingdom; Georgia Institute of Technology, United States of America

## Abstract

Genome-wide association studies have identified hundreds of loci for type 2 diabetes, coronary artery disease and myocardial infarction, as well as for related traits such as body mass index, glucose and insulin levels, lipid levels, and blood pressure. These studies also have pointed to thousands of loci with promising but not yet compelling association evidence. To establish association at additional loci and to characterize the genome-wide significant loci by fine-mapping, we designed the “Metabochip,” a custom genotyping array that assays nearly 200,000 SNP markers. Here, we describe the Metabochip and its component SNP sets, evaluate its performance in capturing variation across the allele-frequency spectrum, describe solutions to methodological challenges commonly encountered in its analysis, and evaluate its performance as a platform for genotype imputation. The metabochip achieves dramatic cost efficiencies compared to designing single-trait follow-up reagents, and provides the opportunity to compare results across a range of related traits. The metabochip and similar custom genotyping arrays offer a powerful and cost-effective approach to follow-up large-scale genotyping and sequencing studies and advance our understanding of the genetic basis of complex human diseases and traits.

## Author Summary

Recent genetic studies have identified hundreds of regions of the human genome that contribute to risk for type 2 diabetes, coronary artery disease and myocardial infarction, and to related quantitative traits such as body mass index, glucose and insulin levels, blood lipid levels, and blood pressure. These results motivate two central questions: (1) can further genetic investigation identify additional associated regions?; and (2) can more detailed genetic investigation help us identify the causal variants (or variants more strongly correlated with the causal variants) in the regions identified so far? Addressing these questions requires assaying many genetic variants in DNA samples from thousands of individuals, which is expensive and timeconsuming when done a few SNPs at a time. To facilitate these investigations, we designed the “Metabochip,” a custom genotyping array that assays variation in nearly 200,000 sites in the human genome. Here we describe the Metabochip, evaluate its performance in assaying human genetic variation, and describe solutions to methodological challenges commonly encountered in its analysis.

## Introduction

Recent data emerging from theoretical models [1], [2] and empirical observation through genome-wide association studies (GWAS) (for example [3], [4]) demonstrate that hundreds of genetic loci contribute to complex traits in humans. These data prompt two questions: (1) can additional genetic loci be identified by follow-up of the most significantly associated variants after initial GWAS meta-analysis? and (2) can further investigation via genetic fine-mapping refine association signals at established genetic loci? Systematically addressing these two questions should help improve understanding of the genetic architecture of complex traits and their shared genetic determinants, and suggest hypotheses and disease mechanisms that can be tested in functional experiments or model systems [5].

Addressing these two questions requires genotyping thousands of individuals at many genetic markers. For most currently available genotyping technologies, this kind of characterization is cost-prohibitive. To address this need in the context of type 2 diabetes, coronary artery disease and myocardial infarction, and quantitative traits related to these diseases, we designed the Metabochip, a custom genotyping array that provides accurate and cost-effective genotyping of nearly 200,000 single nucleotide polymorphisms (SNPs) chosen based on GWAS meta-analyses of 23 traits (Table 1). Metabochip SNPs were selected from the catalogs developed by the International HapMap [6] and 1000 Genomes [7] Projects, allowing inclusion of SNPs across a wide range of the allele frequency spectrum. These included 63,450 SNPs to follow-up the top ∼5,000 or ∼1,000 (see Methods) independent association signals for each of the 23 traits, 122,241 SNPs to fine-map 257 loci which showed genome-wide significant evidence for association with one or more of the 23 traits, and 16,992 SNPs chosen for a variety of other reasons (see Methods and Table 2). In designing the array, we sought to maximize assay success rates as well as the number of variants that could be assayed; Illumina custom arrays include a fixed number of “beads” and some sites can be assayed with a single bead while others require two [8].

**Table 1:** Summary of Metabochip SNPs by trait: Fine-mapping and replication.

Consortium	Trait Name	Fine Mapping			Replication SNPs
		# Loci	Size (Mb)	# SNPs	
**Tier 1**					
DIAGRAM	Type 2 Diabetes	34	6.56	16,717	5,057
CARDIoGRAM	MI and CAD	30	9.60	19,558	6,485
Lipids	HDL Cholesterol	23	4.62	12,150	5,024
	LDL Cholesterol	21	4.06	9,981	5,060
	Triglyceride	20	4.68	9,784	5,057
GIANT	Body Mass Index	24	7.48	18,211	5,055
	Waist-to-Hip Ratio*	15	2.25	5,464	5,056
MAGIC	Fasting Glucose	19	5.05	13,644	5,058
ICBP	Diastolic Blood Pressure	20	8.34	13,239	5,060
	Systolic Blood Pressure	21	6.01	10,641	5,059
QT-IGC	QT Interval	18	4.08	10,910	5,041
**Tier 2**					
DIAGRAM	T2D Age of Diagnosis	0	0.00	0	1,039
	T2D Early Onset	0	0.00	0	1,040
HaemGen	Mean Platelet Volume	0	0.00	0	657
	Platelet Count	0	0.00	0	577
	White Blood Cell	0	0.00	0	598
Lipids	Total Cholesterol	0	0.00	0	941
Body Fat	Body Fat Percentage	0	0.00	0	1,035
GIANT	Height	0	0.00	0	1,050
	Waist Circumference*	2	0.50	1,374	1,048
MAGIC	2-Hour Glucose	3	0.61	1,249	1,038
	Glycated Hemoglobin	5	0.46	2,181	1,045
	Fasting Insulin	2	0.67	1,309	1,046
TOTAL	With Redundancy	257	64.97	146,453	68,126
	Unique Regions/SNPs	257	45.52	122,241	63,450

*SNP counts are numbers of SNPs successfully manufactured on the Metabochip array.*

* *Waist-to-hip ratio and waist circumference were adjusted for body mass index.*

**Table 2:** Summary of Metabochip SNPs by SNP category.

SNP Category	Chosen for Array	Passed Manufacture	Among 67 HapMap samples		
			>95% Called	MAF>0	MAF<.05
Replication	66,130	63,450 (95.9%)	61,386 (96.7%)	60,585 (98.7%)	6,121 (10.1%)
Fine-Mapping	139,877	122,241 (87.4%)	116,779 (95.5%)	92,731 (79.4%)	37,552 (40.5%)
Prior Trait Association	2,210	2,116 (95.7%)	2,043 (96.5%)	2,039 (99.8%)	235 (11.5%)
CNP tags	6,888	6,626 (96.2%)	6,250 (94.3%)	6,160 (98.6%)	941 (15.3%)
MHC	3,203	2,909 (90.8%)	2,550 (87.7%)	2,537 (99.5%)	185 (7.3%)
Mitochondrial	144	135 (93.8%)	102 (75.6%)	66 (64.7%)	28 (42.4%)
Chromosome X/Y	112	107 (95.5%)	106 (99.1%)	104 (98.1%)	0 (0%)
Fingerprint	46	43 (93.5%)	40 (93.0%)	40 (100%)	0 (0%)
Wildcard	5,323	5,056 (95.0%)	4,847 (95.9%)	4,108 (84.8%)	493 (12.0%)
TOTAL (without redundancy)	217,695	196,725 (90.4%)	188,395 (95.8%)	163,107 (86.6%)	44,967 (27.6%)

*Numbers in parenthesis represents the proportion of the SNPs in the previous column. A SNP may fall into multiple categories.*

Here, we describe Metabochip array design, and evaluate performance of the array in common genetic analysis steps, including quality control steps such as genomic control calculations, identification of related individuals, and fine-mapping of known disease susceptibility loci. Our results provide practical guidance to investigators and show that for fine-mapping loci the Metabochip provides much greater resolution than prior GWAS arrays.

## Methods

### Core Features of the Metabochip: Traits and SNPs

The Metabochip was designed by representatives of the Body Fat Percentage [9], CARDIoGRAM (coronary artery disease and myocardial infarction) [10], DIAGRAM (type 2 diabetes) [11], GIANT (anthropometric traits) [3], [12], [13], Global Lipids Genetics (lipids) [4], HaemGen (hematological measures) [14], ICBP (blood pressure) [15], MAGIC (glucose and insulin) [16]–[18], and QT-IGC (QT interval) [19], [20] GWAS meta-analysis consortia. The array is comprised of SNPs selected across two tiers of traits (Table 1). Tier 1 is comprised of eleven traits deemed to be of primary interest: type 2 diabetes (T2D), fasting glucose, coronary artery disease and myocardial infarction (CAD/MI), low density lipoprotein (LDL) cholesterol, high density lipoprotein (HDL) cholesterol, triglycerides, body mass index (BMI), systolic and diastolic blood pressure, QT interval, and waist-to-hip ratio adjusted for BMI (WHR). Tier 2 is comprised of twelve traits of secondary interest: fasting insulin, 2-hour glucose, glycated hemoglobin (HbA1c), T2D age of diagnosis, early onset T2D (diagnosis age<45 years), waist circumference adjusted for BMI, height, body fat percentage, total cholesterol, platelet count, mean platelet volume, and white blood cell count.

**Figure 1:**
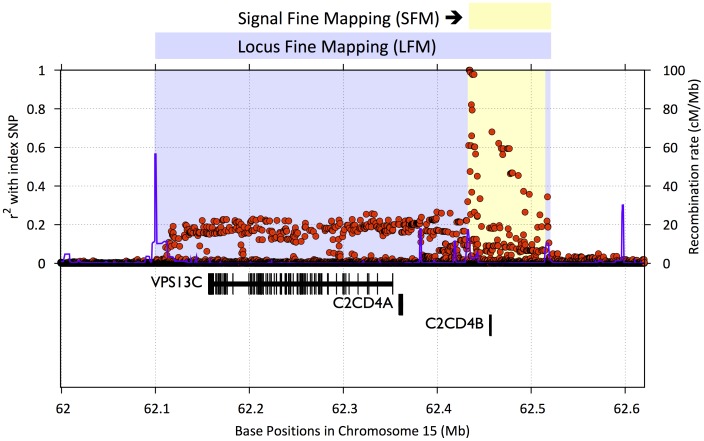
Example of signal fine mapping (SFM) and locus fine mapping (LFM) regions. A SFM region seeks to map the initial association signal. SFM regions were designed using linkage disequilibrium (LD) r^2^ estimates from the 1000 Genomes Project and HapMap CEU data. Initial boundaries were determined by identifying all SNPs satisfying r^2^≥.5 with the index SNP, and then expanded to the nearest flanking recombination hotspot, but stopped if there was no hotspot nearby. LFM regions (blue) were similarly designed but expanded to capture functional units of interest such as nearby coding genes. The figure plots LD r^2^ for SNPs (red dots) within the region and recombination rate (blue lines) as a function of position on the chromosome. Gene positions and structures are displayed in the lower panel. MI = myocardial Infarction; CAD = cardiovascular disease; HDL = high-density lipoprotein; LDL = low-density lipoprotein; T2D = type 2 diabetes.

We included three design classes of SNPs on the Metabochip (Table 2):

1. Replication SNPs: ∼5,000 (Tier 1) or ∼1,000 (Tier 2) SNPs were selected to follow-up the top independent association signals from the largest available GWAS meta-analysis for each of the 23 traits (Supplementary Table S1).
2. Fine-mapping SNPs: SNPs were selected from the catalogs of the International HapMap Project [6] and the August 2009 release of the 1000 Genomes Project [7] to fine-map 257 loci associated at genome-wide significance (P<5×10^−8^) in preliminary analyses of one or more of the 23 traits (See Figure 1, Supplementary Table S2 and S3, and Supplementary Text for details).
3. Other SNPs: These were comprised of independent SNPs for which genome-wide significant associations had been reported for any trait, SNP tags for copy number polymorphisms (CNPs), the MHC region, and the mitochondrial genome, fingerprint SNPs from GWA array products, a set of chromosome X and Y markers for sex verification, and “wild-card” SNPs based on consortium-specific hypotheses and interests (for example, based on a known pathway or early deep-sequencing studies). A detailed description of how SNPs were selected in each of these categories can be found in the Supplementary Text [21]–[25].

In total, 217,695 SNPs were chosen for the array (Table 2). 20,970 SNPs (9.6%) failed during the assay manufacturing process, resulting in 196,725 SNPs available for genotyping. A summary file annotating each Metabochip SNP with ascertainment criteria, SNP assay, a list of unintended duplicate SNPs (Supplementary Table S4), and reference strand orientation for alleles is provided at http://www.sph.umich.edu/csg/kang/MetaboChip/.

### Data Generation and Quality Control (QC)

We evaluated the utility of the Metabochip and accuracy of its genotype calls in three sample sets: (1) 15,896 northern European individuals from the FUSION, METSIM, HUNT, Tromsø, and Diagen studies [26]–[30] together with 67 HapMap samples genotyped at least two times each and called using Illumina GenomeStudio software by re-clustering these data; (2) 6,614 Sardinian individuals organized in 1,243 extended families from the SardiNIA study [31], [32] called by GenomeStudio software using default cluster data; and (3) 9,715 Nordic individuals from the Malmø Preventive Project, the Scania Diabetes Registry, and the Botnia Study [33]–[35] genotyped using a modified version of the BIRDSEED genotype calling algorithm [36].

We applied standard SNP- and sample-based QC filters based on call rate, Hardy-Weinberg equilibrium deviations, duplicate genotype inconsistencies, and failures of Mendelian inheritance; in the Nordic sample, we also carried out checks based on plate-specific characteristics. These filters resulted in final data sets of 163,222 polymorphic SNPs genotyped in 67 HapMap samples, 142,812 polymorphic SNPs genotyped in 6,164 Sardinians, and 179,165 polymorphic SNPs genotyped in 8,473 Nordic individuals.

### Statistical Analysis Using Metabochip: Genomic Control, PCA, and Kinship Estimation

Since Metabochip SNPs were selected to be associated with our 23 traits of interest, performing genomic control correction [37] requires some care. To select a set of (near)-independent SNPs that are not associated with an analysis trait of interest, we focused on SNPs selected to replicate signals unrelated to the trait of interest (for example, QT interval SNPs for a T2D association analysis), also removing SNPs within 250 kb of SNPs previously associated with the trait of interest, and then LD-pruning the remaining SNPs so that no SNP pair is in strong LD (r^2^>.3).

To estimate kinship coefficients or to correct for population stratification using principal components analysis (PCA) or multidimensional scaling (MDS) covariates, we require SNPs that are not too rare and are not in strong pairwise LD. We found that taking SNPs with MAF>.05 and LD-pruning them so that no SNP pair has r^2^>.3 works well for PCA and MDS (data not shown). The same subset of SNPs can be used for pairwise IBD estimation using the maximum-likelihood method of Milligan [38] implemented in PLINK [39] or the variance-components method of Balding and Nichols [40] implemented in EMMAX [41].

### Imputation Preparation and Evaluation

We carried out genotype imputation in the Sardinian data. We imputed variants observed in a reference set of 280 Europeans from the August 2010 1000 Genomes Project data into: (a) 6,164 individuals genotyped on the Metabochip [32], (b) 1,097 individuals genotyped on the Affymetrix 6.0 array, and (c) 1,412 individuals genotyped on the Affymetrix 500 K array [42]. We evaluated mean estimated r^2^ within fine-mapping regions using minimac ([43]; www.genome.sph.umich.edu/wiki/minimac), and empirically compared the imputation quality using the published Sanger sequencing data in five fine mapping loci [32]. In addition, we evaluated mean estimated r^2^ across different continental populations by leaving one individual out from the 1000 Genomes reference panel and imputing them using markers present in each platform across the fine mapping regions and a 1 Mb window flanking each region. We also compared association power obtained by imputation into GWAS and Metabochip samples in Metabochip fine-mapping regions by comparing LDL cholesterol association evidence in 2,342 of these individuals genotyped using both the Metabochip and one of the Affymetrix arrays.

## Results

### Evaluation of Array Design and Genotype Quality

Of 217,695 SNPs chosen for the Metabochip across all design categories, 196,725 (90.4%) were successfully manufactured on the array (Table 2). The 48,846 previously manufactured SNPs had higher success rate (95.4%) than the 168,849 new SNP assays (88.7%). Illumina design score was predictive of the quality of manufactured SNP assays. For example, 25% of SNPs with design score<0.6 failed to produce genotype calls due to poor clustering of the intensity data, compared to 3.1% of SNPs with design score between 0.6 and 1.0 (Supplementary Figure S1).

We evaluated genotype calling accuracy for 67 HapMap samples genotyped multiple times using three different calling strategies: (a) Illumina GenomeStudio with reclustering the intensity data using >15,000 samples; (b) Illumina GenomeStudio based on default clusters provided by Illumina; and (c) GenoSNP [44], which calls genotypes based on a within-sample-between-markers analysis of intensity data rather than a between-sample-within-marker analysis.

The large majority of Metabochip SNPs yielded high quality genotypes. For the 67 HapMap samples called using GenomeStudio with reclustering, only 8,344 (4.2%) of the 196,725 SNP assays had genotype call rates <95%, while another 25,958 SNPs (13.2%) were monomorphic. Using GenomeStudio and default clusters, these numbers were 12,131 (6.2%) and 25,311 (12.9%), while using GenoSNP, they were 18,107 (9.2%) and 25,532 (13.0%).

Using GenomeStudio with reclustering, genotype concordance between Metabochip genotypes for duplicate pairs was 99.998% overall and 99.990% for heterozygotes. Comparing Metabochip genotypes to HapMap 3 genotypes for the 59,935 SNPs in common, genotype concordance was 99.93% overall and 99.84% for heterozygotes, similar to the 99.87% Mendelian consistency rate reported in the HapMap3 data [45]. We observed similar concordance rates for these sample sets using the Illumina caller with default clusters (99.93% overall, 99.84% for heterozygotes), or using GenoSNP [44] (99.85% overall, 99.81% for heterozygotes).

Genotype concordance for less common variants was slightly lower than for common variants. For example, among the singleton SNPs in the 67 HapMap samples, 98.9% of heterozygous genotypes were concordant with HapMap3 for the two GenomeStudio call sets and 97.8% for the GenoSNP set. Heterozygous genotype concordances for singleton SNPs between duplicate pairs were 99.76%, 99.70%, and 99.83% for the three call sets.

### Frequency Spectrum and Coverage

**Figure 2:**
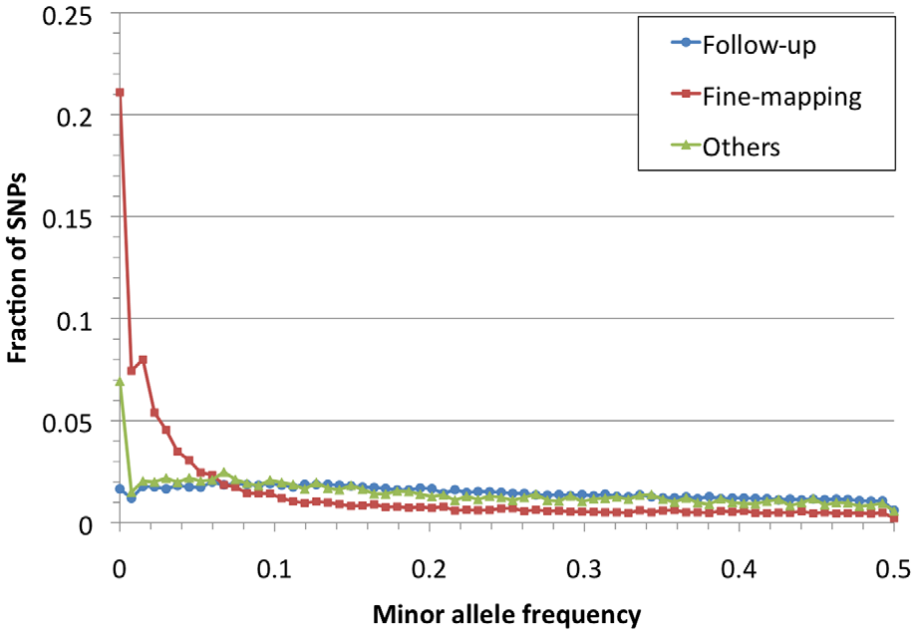
Allele frequency spectrum for Metabochip SNPs by design category. Blue dots, red squares, and green triangles display fractions of replication, fine-mapping, and all other SNPs (see Table 2) in each of the tabulated minor allele-frequency bins. CNP = copy number polymorphism.

We evaluated the allele frequency spectrum for Metabochip SNPs in the 67 HapMap samples (Figure 2). Mean MAF of Metabochip SNPs was .152 overall, .109 among fine-mapping SNPs, and .224 among replication SNPs. Among these three SNP sets, 38%, 53%, and 12% of SNPs had MAF<.05, and 14%, 21%, and 2% were monomorphic.

**Figure 3:**
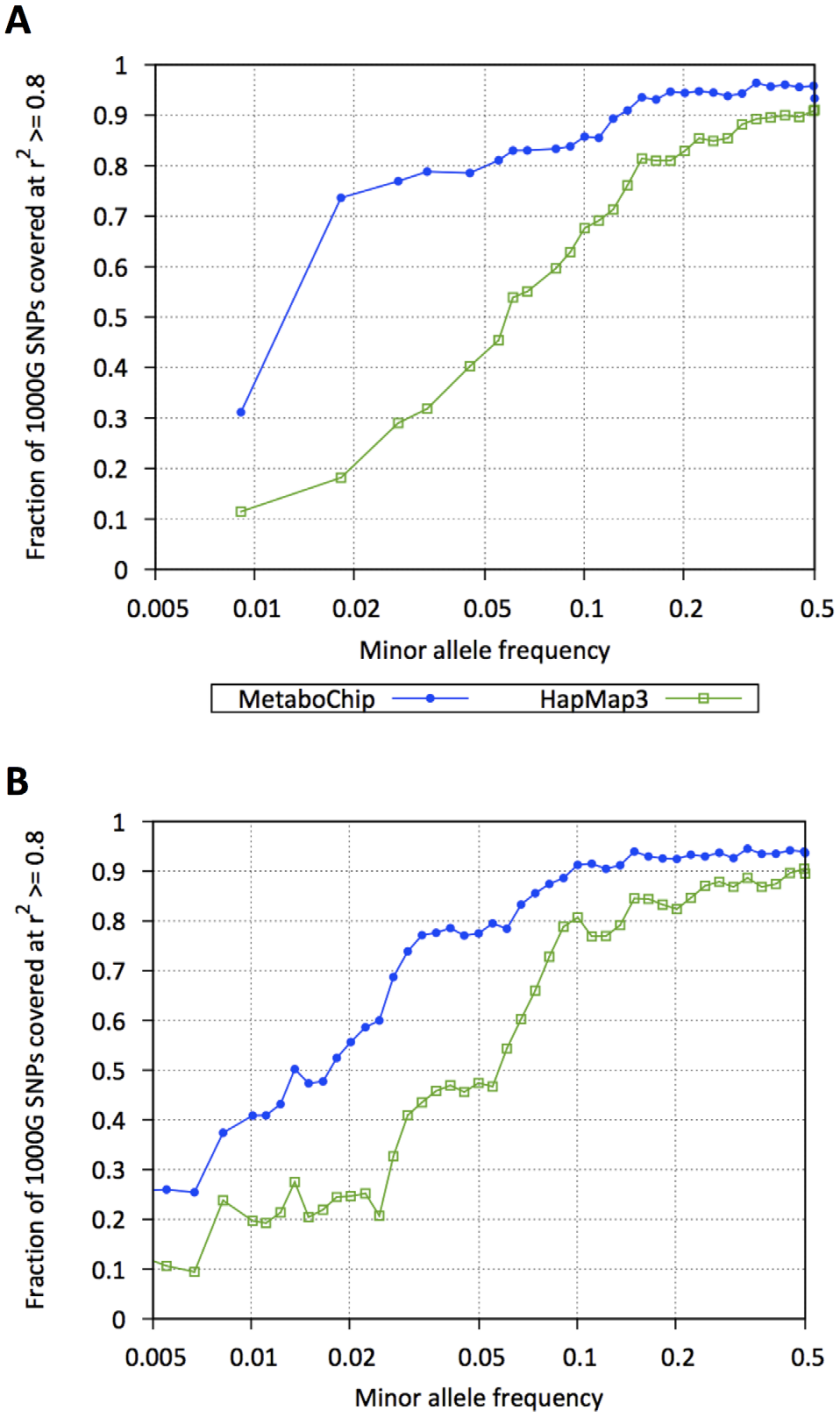
Coverage of 257 Metabochip fine-mapping regions. Fraction of 1000 Genomes Project SNPs in strong linkage disequilibrium (r^2^≥.8) with HapMap 3 (green squares) or Metabochip (blue dots) SNPs as a function of minor allele frequencies: (A) 1000 Genomes Pilot 1 SNPs, (B) 1000 Genomes Phase 1 SNPs (May 2011 release).

Within the 257 fine-mapping regions (45.52 Mb), 109,855 SNPs were catalogued by the 1000 Genomes Project [7] pilot studies and 240,805 SNPs are in the current Phase 1 release (as of November 2011). Of these, 122,241 fine-mapping SNPs were genotyped on the Metabochip (Supplementary Table S2). In the 1000 Genomes European samples, Metabochip SNPs tag 82.0% and 54.5% of all Pilot and Phase 1 1000 Genomes variants in these regions at r^2^≥.8, compared to 61.3% and 40.3% coverage using HapMap 3 SNPs (Figure 3). Among SNPs with MAF<.05, Metabochip SNPs tag 61.9% and 33.8% at r^2^≥.8, compared to 24.3% and 17.0% using HapMap 3. Using genotype imputation, we can impute 82% of 1000 Genomes Phase 1 European SNPs with MAF>0.5% with an estimated r^2^≥0.8.

### Genotype Imputation within the Metabochip Fine-Mapping Regions

**Figure 4:**
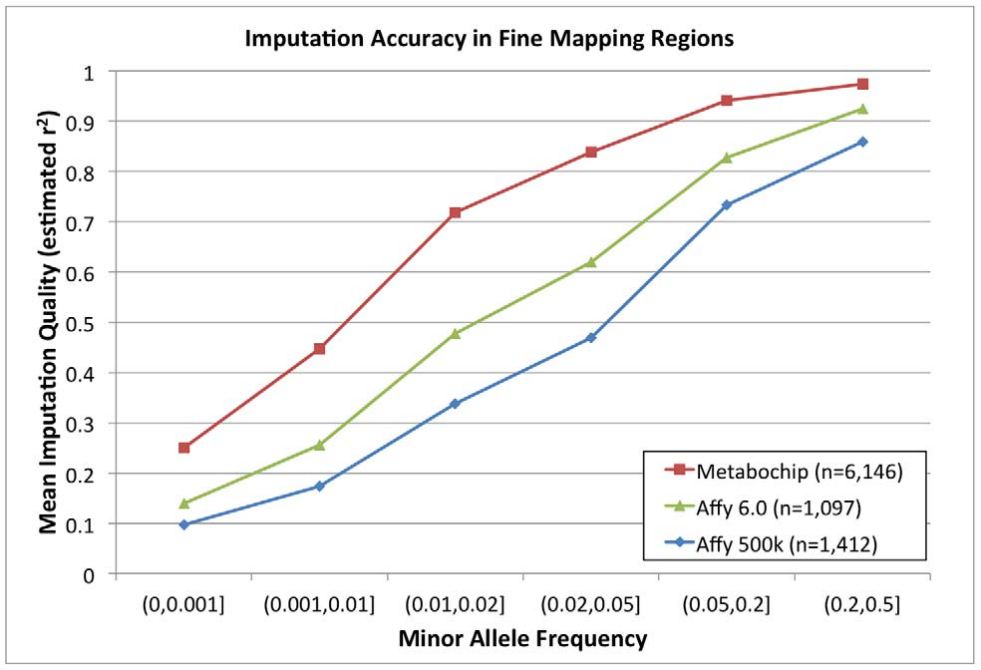
Imputation accuracy (estimated r^2^) in fine mapping regions. Imputation accuracy for differing numbers of Sardinian individuals as measured by estimated r^2^ value across the 257 Metabochip fine mapping regions for Metabochip (red squares), Affymetrix 6.0 GWAS SNPs (green triangles), and Affymetrix 500 k GWAS SNPs (blue circles) as a function of minor allele frequency bin.

We next investigated accuracy of genotype imputation into the 257 Metabochip fine-mapping regions using the 280 Europeans from 1000 Genomes Project [7] as reference set and the 6,164 individuals in the Sardinian Metabochip sample as target. Figure 3 displays estimated r^2^ values in the Metabochip fine-mapping regions as a function of MAF. Also displayed are estimated r^2^ values for SNPs in these regions using the 280 European 1000 Genomes project samples as reference set and 1,412 Sardinians genotyped on the Affymetrix 500 K and 1,097 Sardinians genotyped on the Affymetrix 6.0 chips as targets. Imputation accuracy into the Sardinian Metabochip sample is greater in all allele frequency ranges than for the samples genotyped using the GWAS arrays. For example, among SNPs with .02≤MAF<.05, mean estimated r^2^ for the Affymetrix 500 K, Affymetrix 6.0, and Metabochip samples were .47, .62, and .84, respectively (Figure 4). The improved imputation accuracy for Metabochip compared to GWAS array is primarily due to increased marker density of the Metabochip in these regions.

Imputation quality in the Metabochip fine-mapping regions using Metabochip is also improved for non-European individuals compared to imputation using GWAS platforms. Using a leave-one-sample-out approach, we evaluated the average r^2^ from the 1000 Genomes reference panel into Affymetrix 500 k, Affymetrix 6.0, and Metabochip. For example, among SNPs with .02<MAF<.05, mean estimated r^2^ across European individuals for the chips were .78, .83, and .93, respectively. For individuals with African ancestry, corresponding values were .78, .85, and .94, and for individuals of Asian ancestry, they were .67, .72, and .89 (Supplementary Figure S2). The fact that imputation of rare variants in African ancestry populations is more accurate than in European populations is probably explained by noting that – in the short regions evaluated here – there will be only a limited number of common variant haplotypes in Europeans and, in some cases, these will not effectively tag specific rare variants. In African populations, with a larger variety of rare haplotypes, it is more likely (relative to Europeans) that at least one haplotype will capture rare variants of interest.

In addition, we empirically evaluated the quality of experimentally determined and imputed SNPs within the five fine mapping regions by comparing individual genotypes with those obtained by Sanger sequencing. For 126 SNPs evaluated, the average r^2^ in analyses based on the Affymetrix 500 k and 6.0 arrays was .46 and .55, respectively. Analyses based on Metabochip showed average r^2^ = .79. Focusing on 48 SNPs that were imputed in all three analyses, the average r^2^ was .31 (Affymetrix 500 K), .41 (Affymetrix 6.0), and .57 (Metabochip) (Supplementary Figure S3).

### High-Resolution Association Analysis within Metabochip Fine-Mapping Regions

**Figure 5:**
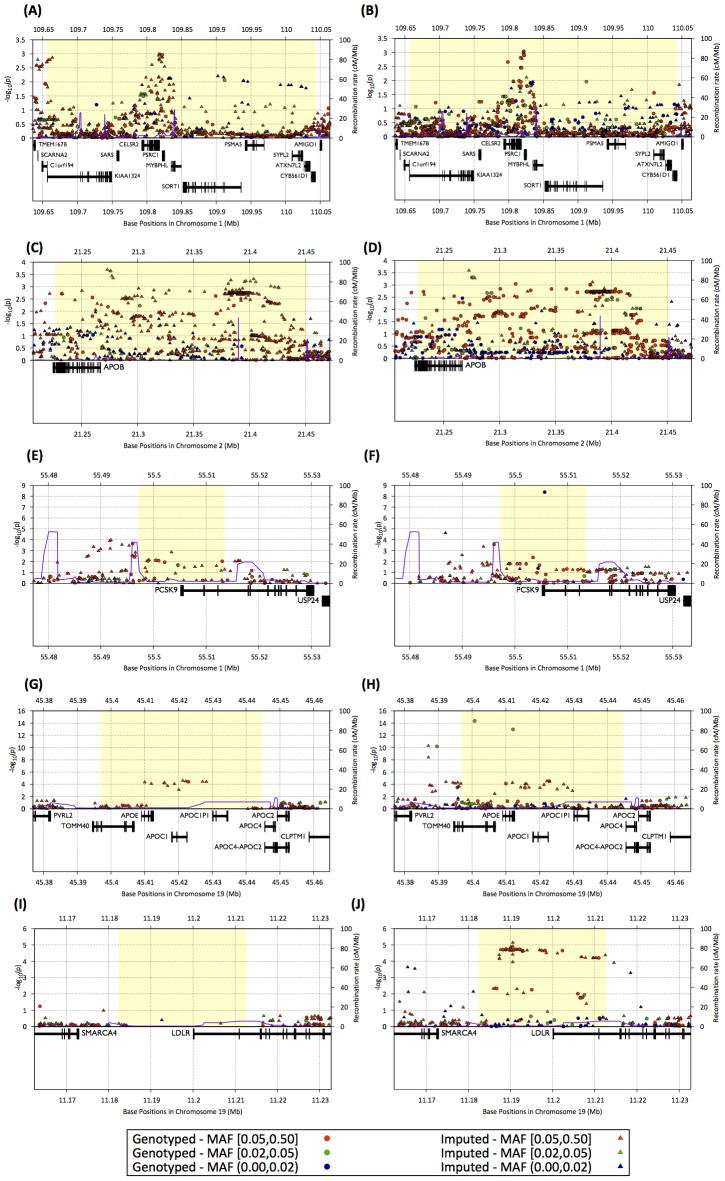
Regional association plots for LDL cholesterol association in the SardiNIA study. Association plots for a study of 2,432 Sardinian individuals for five Metabochip fine-mapping regions using 1000 Genomes data as reference set and Affymetrix genotypes (left panels : A,C,E,G,H) or Metabochip genotypes (right panels : B,D,F,H,J) as target sets. The figures plot −log_10_ of the association p-value within the region and recombination rate (blue lines) as a function of position on the chromosome. Blue, green, and red dots and triangles indicate genotyped and imputed SNPs with minor allele frequencies less than 0.02, greater than or equal 0.02 and less than 0.05, and greater than or equal 0.05, respectively. Gene positions and structures are displayed in the lower panel.

To compare the power and resolution for association testing in the Metabochip fine-mapping regions to that of standard GWAS arrays, we revisited the LDL cholesterol association analysis from the SardiNIA study [32] in 2,342 individuals genotyped for both Metabochip and an Affymetrix (6.0 or 500 k) GWAS chip. Here, we focus on five of the six most strongly associated loci from Willer et al. [46], in and around *PCSK9*, *LDLR*, *APOE*/*APOC1*/*APOC2*, *SORT1*, and *APOB* (Figure 5A–J), all of which were designated for locus fine mapping by the Global Lipids Genetics Consortium.

In the *SORT1* and *APOB* regions, the peak association signals for the two data sets are similar (Figure 5A–D). For *PCSK9*, *LDLR*, and *APOE*/*APOC1*/*APOC2*, Metabochip based analysis resulted in considerably stronger association signals. For *PCSK9* and *APOE*/*APOC1*/*APOC2*, the most strongly associated variants were low-frequency SNPs (MAF = 1.1% for *PCSK9*, MAF = 3.4% for *APOE*) that were directly genotyped on the Metabochip but not on the Affymetrix chips (Figure 5E–J). Although the signals from common variants are similar, the peak SNPs were not imputed accurately in the Affymetrix data (estimated r^2^ = .04 and .08, respectively). Within the *LDLR* region, there are 165 SNPs in the 1000 Genomes European panel. None of these SNPs are on the Affymetrix chips and only eight could be imputed at estimated r^2^≥.3 using the Affymetrix data; the locus is also hard to impute using HapMap 2 as a reference, with the peak association signals corresponding to r^2^ of ∼.40. In contrast, 36 of the 165 SNPs were directly genotyped in Metabochip, and 122 were imputed at estimated r^2^≥.3. As a result, imputation into the Metabochip data resulted in a substantial association signal (p = 7.3×10^−6^), while for the Affymetrix data, p>.02 at all markers (Figure 5I–J). These results demonstrate that dense genotyping may substantially improve imputation accuracy, increasing association power even for common variants.

### Performing Standard Statistical Analyses Using Metabochip Genotype Data

We carried out kinship estimation between pairs of individuals and calculated genotype-based principal components for inclusion as covariates in genetic association analysis using all Metabochip SNPs that passed QC, and then using the pruned subset of SNPs described in the Methods section. When using all QC-passing SNPs, estimates of pairwise kinship coefficients in the Sardinia sample had inflated variance (Supplementary Table S5), and kinship coefficient estimates for the Nordic sample calculated using PLINK suggested (incorrectly) that essentially all pairs of individuals were related (Supplementary Figure S4). For each analysis, using the pruned set of SNPs gave sensible results, reducing variance in estimated kinship coefficients in the Sardinia sample and removing the artifactual estimates of close relatedness in the Nordic sample.

Because many Metabochip SNPs were included specifically due to prior evidence for association of T2D, CAD/MI and related traits, controlling for potential population stratification in Metabochip analysis requires some care. Not surprisingly, carrying out T2D association analysis in the Nordic sample on all SNPs passing QC without inclusion of genotype-based principal components resulted in a large genomic control inflation factor (λ_GC_ = 1.44). Including all SNPs that passed QC to estimate principal components (PCs), and then including those PCs as covariates in the association analysis gave reduced but still substantial inflation (λ_GC_ = 1.13). When we instead estimated test statistic inflation based only on the 3,772 LD-pruned QT interval replication SNPs (not expected to associate with T2D) we obtained a genomic control inflation factor near unity (λ_GC_ = 1.01).

### Assessing Overlap among SNPs across Traits

We were interested whether the replication SNP sets submitted by the GWAS consortia for the different traits showed more or less overlap than expected by chance. To address this question, we counted the number of SNPs in common across pairs of traits, and used simulation to test whether the observed overlaps were different than expected under the null hypothesis of genetic independence of pairs of traits (Supplementary Table S6). Not surprisingly, we observed substantial SNP set overlaps (and greater than expected assuming independence) for multiple pairs of correlated traits, notably SBP and DBP (38% proportion of maximum possible overlap), HDL and TG (17%), and TC and LDL (87%). We also observed substantial genetic overlap (4%) between LDL and SBP, which are nearly uncorrelated traits. Overall, we observed an excess of nominally significant SNP set overlaps, consistent with (but in no way proof of) the hypothesis a shared genetic etiology between these cardiometabolic traits.

## Discussion

We designed the Metabochip, a custom genotyping array for replication of the top association signals from the largest available GWAS meta-analysis for 23 T2D and CAD/MI related traits and for fine-mapping 257 genome-wide significant association signals for 15 of these traits (Table 1). The Metabochip also includes a set of SNPs representing genome-wide significant associations across a range of human traits; SNPs that tag known copy number polymorphisms, the MHC, and mitochondrial variants; X and Y chromosome SNPs for sex verification, fingerprint SNPs for sample tracking, and “wildcard” SNPs selected by the participating GWAS consortia (Table 2). The array has already been genotyped on DNA samples from hundreds of thousands of individuals and preliminary analyses across the contributing GWAS consortia have identified hundreds of new genome-wide association signals (manuscripts being prepared by each of the consortia).

In designing the Metabochip, 90.4% of chosen SNPs were successfully designed and manufactured onto the array, and of these, ∼82% passed QC filters in our three example studies, resulting in very complete coverage of variation in our 257 fine-mapping regions. Of course, as time passes and catalogs of SNPs expand, potential shortcomings in coverage should become apparent. Currently, coverage of 1000 Genomes Pilot Study European SNPs in the fine-mapping regions is 82.0% at a tagging threshold of r^2^≥.8. Coverage of Phase 1 European SNPs in these regions is 54.5%, and the number increases to 73.7% for SNPs at MAF>0.5%. Using genotype imputation, we can impute 82% of 1000 Genomes Phase 1 European SNPs with MAF>0.5% with estimated r^2^≥0.8. The resulting data are of high quality, with 99.99% duplicate consistency in heterozygotes and 99.77% Mendelian consistency in heterozygotes in our studies. Further, Metabochip fine-mapping regions provide an excellent target for genotype imputation from relevant reference sets, and in our experience can provide more complete coverage than provided by standard HapMap-based GWAS arrays (Figure 3) for both common and less common variants.

A key decision in the fine-mapping of any GWAS signal concerns the size of the region where genetic variation will be examined exhaustively. In designing the Metabochip, we focused on relatively small regions surrounding each lead SNP – these included all variants in strong linkage disequilibrium (r^2^>.5) and a small shoulder extending .02 cM beyond that (typically, ∼20 kb). This decision was informed by the observation that, in cases where GWAS signals and Mendelian disease loci overlap, they are typically very close together (typically within ∼10 kb of each other and nearly always within <100 kb; see [4] for a discussion of the issue), although there are exceptions to this rule (see [47], for example).

Within each fine-mapping region, we selected variants identified by the HapMap consortium and early analyses of the 1000 Genomes Consortium data. The 1000 Genomes Project and other sequence based catalogs of genetic variation are now more extensive that at the time of array design, but (as noted above) our analyses show that the SNPs selected for inclusion in the Metabochip form a useful reagent for genotyping imputation – not only for the imputation of newly discovered SNPs in the fine-mapping regions (see above) but also for the imputation of other types of variants, such as indel polymorphisms, that have become part of newer 1000 Genomes Project analyses (unpublished data).

Several other design choices for Metabochip were to some degree arbitrary: which traits to include; balance in numbers of SNPs for replication, fine mapping, and other purposes; and how to prioritize among SNPs available for each purpose. Were we to design a similar chip now, we would take advantage of the now available more extensive and deeply annotated SNP catalogs. In addition, we would likely include a set of randomly ascertained SNPs to facilitate analysis that control for population structure and other artifacts. Finally, with empirical evidence from this and other projects on the relationship between SNP design score and empirical probability of successful design, we would likely replace design score by probability of successful design. This approach would likely result in even higher call rates.

Because Metabochip SNPs are highly enriched for trait-associated SNPs and >60% are clustered in the ∼1.5% of the genome that comprises the fine-mapping regions, Metabochip genotype data present some challenges to standard analyses such as relationship estimation, principal components analysis, and genomic control determination. However, as we demonstrated, these challenges can be overcome by focusing on replication SNPs expected to be unrelated to the trait of interest. An alternative approach is to use SNPs that were not associated with the trait(s) of interest in the corresponding GWAS (for example, p-value>.50 for all such traits) and then to LD-prune the resulting set of SNPs to identify a near-independent set. An alternative that is also worthy of investigation in the analysis of case-control samples is the application of principal component factor loadings derived from a controls-only analysis to the combined sample of cases and controls. When this last alternative is considered, it is important to check that PCA axes derived from controls represent all relevant ancestries present in cases. The design of the array, focused on replication and fine-mapping and selecting SNPs from early releases of the HapMap and 1000 Genomes Projects, resulted in a highly non-random ascertainment of SNPs. Thus, we cannot recommend use of Metabochip SNPs for population genetic analyses that rely on unbiased, and/or comprehensive ascertainment schemes for SNPs.

The need for follow-up genotyping is a frequent requirement of GWAS and sequencing studies of complex human traits. Approaching array design in a coordinated fashion across related studies and traits can be particularly cost-effective, since per array costs often drop dramatically with increasing numbers of individuals to be genotyped, and (given sufficient numbers of individuals) may increase only modestly with increasing numbers of SNPs. For example, a custom chip designed to genotype the ∼22,000 DIAGRAM-selected type 2 diabetes Metabochip SNPs in the ∼80,000 individuals genotyped on Metabochip by the DIAGRAM consortium studies would have cost ∼$55 compared to the Metabochip cost of $39, delivering only 1/9 as many genotypes at >40% greater cost. Furthermore, examining the association between SNPs tentatively associated with one trait for other related traits can also be informative, highlighting pleiotropy across related traits and helping discover new association signals; for example, two of the ten novel type 2 diabetes loci identified to date by Metabochip analysis by the DIAGRAM consortium were placed on Metabochip for other traits [48]. In the case of the Metabochip, which is less expensive than many smaller trait specific arrays, this opportunity to collect more information and investigate the effects of SNPs associated with other traits actually comes with reduced costs (compared to trait specific arrays), although with the need to organize across multiple consortia and to share the number of SNPs that can be cost-effectively genotyped. The “Immunochip” [49] follows this same paradigm and supports genotyping of ∼200,000 SNPs identified on the basis of GWAS meta-analyses for immunological disorders, while the recently designed “exome chip” (Benjamin Neale, Gonçalo Abecasis, personal communication) supports genotyping of ∼250,000 exonic SNPs identified via large-scale exome sequencing studies totaling >12,000 individuals. These and other similar array products represent valuable tools in ongoing efforts to understand the genetic architecture of complex human traits.

## Supporting Information

Figure S1 **Distribution of Illumina design scores by Metabochip SNP category.**
(TIFF)

Figure S2 **Imputation accuracy in fine mapping regions across three continental populations for (A) Europeans (B) Africans, and (C) East Asians.**
(TIFF)

Figure S3 **Empirical concordance between Sanger sequencing data and imputed genotypes.** Empirical r^2^ was evaluated between Sanger sequencing data and imputed genotypes from Metabochip or (A) Affymetrix 500 K SNPs and (B) Affymetrix 6.0 SNPs across five loci in 256 Sardinians.
(TIFF)

Figure S4 **Distribution of estimates of pairwise genome-wide identity-by-descent (IBD) sharing generated by PLINK for all SNPs and for pruned SNPs.**
(TIFF)

Table S1 **Summary of replication SNP submission.**
(EPS)

Table S2 **Summary of fine-mapping regions.**
(EPS)

Table S3 **Summary of SNPs within fine-mapping loci.**
(EPS)

Table S4 **List of unintended duplicated SNPs.**
(EPS)

Table S5 **Estimation of pairwise kinship coefficients.**
(EPS)

Table S6 **Observed count of SNPs in common (upper) between Tier 1 and Tier 2 replication traits submissions and significance of observed overlap (lower).**
(EPS)

Text S1 **Technical details of SNP selection criteria.**
(DOC)

## References

[1] Yang J, Benyamin B, McEvoy BP, Gordon S, Henders AK, et al (2010) Common SNPs explain a large proportion of the heritability for human height. Nat Genet 42: 565–569. 20562875 10.1038/ng.608 PMC3232052

[2] Yang J, Manolio TA, Pasquale LR, Boerwinkle E, Caporaso N, et al (2011) Genome partitioning of genetic variation for complex traits using common SNPs. Nat Genet 43: 519–525. 21552263 10.1038/ng.823 PMC4295936

[3] Allen HL, Estrada K, Lettre G, Berndt SI, Weedon MN, et al (2010) Hundreds of variants clustered in genomic loci and biological pathways affect human height. Nature 467: 832–838. 20881960 10.1038/nature09410 PMC2955183

[4] Teslovich TM, Musunuru K, Smith AV, Edmondson AC, Stylianou IM, et al (2010) Biological, clinical and population relevance of 95 loci for blood lipids. Nature 466: 707–713. 20686565 10.1038/nature09270 PMC3039276

[5] Musunuru K, Strong A, Frank-Kamenetsky M, Lee NA, Ahfeldt T, et al (2010) From noncoding variant to phenotype via SORT1 at the 1p13 cholesterol locus. Nature 466: 714–719. 20686566 10.1038/nature09266 PMC3062476

[6] Frazer KA, Ballinger DG, Cox DR, Hinds DA, Stuve LL, et al (2007) A second generation human haplotype map of over 3.1 million SNPs. Nature 449: 851–861. 17943122 10.1038/nature06258 PMC2689609

[7] The 1000 Genomes Project Consortium (2010) A map of human genome variation from population-scale sequencing. Nature 467: 1061–1073. 20981092 10.1038/nature09534 PMC3042601

[8] Gunderson KL (2006) Steemers FJ (2006) Ren H (2006) Ng P (2006) Zhou L (2006) Whole-genome genotyping. Methods Enzymol 410: 359–376. 16938560 10.1016/S0076-6879(06)10017-8

[9] Kilpeläinen TO, Zillikens MC, Stančákova A, Finucane FM, Ried JS, et al (2011) Genetic variation near IRS1 associates with reduced adiposity and an impaired metabolic profile. Nat Genet 43: 753–760. 21706003 10.1038/ng.866 PMC3262230

[10] Schunkert H, König IR, Kathiresan S, Reilly MP, Assimes TL, et al (2011) Large-scale association analysis identifies 13 new susceptibility loci for coronary artery disease. Nat Genet 43: 333–338. 21378990 10.1038/ng.784 PMC3119261

[11] Voight BF, Scott LJ, Steinthorsdottir V, Morris AP, Dina C, et al (2010) Twelve type 2 diabetes susceptibility loci identified through large-scale association analysis. Nat Genet 42: 579–589. 20581827 10.1038/ng.609 PMC3080658

[12] Heid IM, Jackson AU, Randall JC, Winkler TW, Qi L, et al (2010) Meta-analysis identifies 13 new loci associated with waist-hip-ratio and reveals sexual dimorphism in the genetic basis of fat distribution. Nat Genet 42: 949–960. 20935629 10.1038/ng.685 PMC3000924

[13] Speliotes EK, Willer CJ, Berndt SI, Monda KL, Thorleifsson G, et al (2010) Association analyses of 249,796 individuals reveal 18 new loci associated with body mass index. Nat Genet 42: 937–948. 20935630 10.1038/ng.686 PMC3014648

[14] Soranzo N, Spector TD, Mangino M, Kühnel B, Rendon A, et al (2009) A genome-wide meta-analysis identifies 22 loci associated with eight hematological parameters in the HaemGen consortium. Nat Genet 41: 1182–1190. 19820697 10.1038/ng.467 PMC3108459

[15] Ehret GB, Munroe PM, Rice KM, Bochud M, Johnson AD, et al (2011) Genetic variants in novel pathways influence blood pressure and coronary artery disease risk. Nature 478: 103–109. 21909115 10.1038/nature10405 PMC3340926

[16] Dupuis J, Langenberg C, Prokopenko I, Saxena R, Soranzo N, et al (2010) New genetic loci implicated in fasting glucose homeostasis and their impact on type 2 diabetes risk. Nat Genet 42: 105–116. 20081858 10.1038/ng.520 PMC3018764

[17] Soranzo N, Sanna S, Wheeler E, Gieger C, Radke D, et al (2010) Common variants at 10 genomic loci influence hemoglobin A_1_(C) levels via glycemic and nonglycemic pathways. Diabetes 59: 3229–3239. 20858683 10.2337/db10-0502 PMC2992787

[18] Saxena R, Hivert MF, Langenberg C, Tanaka T, Pankow JS, et al (2010) Genetic variation in GIPR influences the glucose and insulin responses to an oral glucose challenge. Nat Genet 42: 142–148. 20081857 10.1038/ng.521 PMC2922003

[19] Newton-Cheh C, Eijgelsheim M, Rice KM, de Bakker PI, Yin X, et al (2009) Common variants at ten loci influence QT interval duration in the QTGEN Study. Nat Genet 41: 399–406. 19305408 10.1038/ng.364 PMC2701449

[20] Pfeufer A, Sanna S, Arking DE, Müller M, Gateva V, et al (2009) Common variants at ten loci modulate the QT interval duration in the QTSCD Study. Nat Genet 41: 407–414. 19305409 10.1038/ng.362 PMC2976045

[21] Hindorff LA, Sethupathy P, Junkins HA, Ramos EM, Mehta JP, et al (2009) Potential etiologic and functional implications of genome-wide association loci for human diseases and traits. Proc Natl Acad Sci USA 106: 9362–9367. 19474294 10.1073/pnas.0903103106 PMC2687147

[22] de Bakker PI, McVean G, Sabeti PC, Miretti MM, Green T, et al (2006) A high-resolution HLA and SNP haplotype map for disease association studies in the extended human MHC. Nat Genet 38: 1166–1172. 16998491 10.1038/ng1885 PMC2670196

[23] Saxena R, deBakker PI, Singer K, Mootha V, Burtt N, et al (2006) Comprehensive association testing of common mitochondrial DNA variation in metabolic disease. Am J Hum Genet 79: 54–61. 16773565 10.1086/504926 PMC1474138

[24] Van den Ouweland JM, Lemkes HH, Ruitenbeek W, Sandkuijl LA, de Vijlder MF, et al (1992) Mutation in mitochondrial tRNA(Leu)(UUR) gene in a large pedigree with maternally transmitted type 2 diabetes mellitus and deafness. Nat Genet 1: 368–371. 1284550 10.1038/ng0892-368

[25] Poulton L, Luan J, Macaulay V, Hennings S, Mitchell J, et al (2002) Type 2 diabetes is associated with a common mitochondrial variant: evidence from a population-based case-control study. Hum Mol Genet 11: 1581–1583. 12045211 10.1093/hmg/11.13.1581

[26] Schwarz PEH, Towers GW, Fischer S, Govindarajalu S, Schulze J, et al (2006) Hypoadiponectinemia is associated with progression toward type 2 diabetes and genetic variation in the ADIPOQ gene promoter. Diabetes Care 29: 1645–1650. 16801592 10.2337/dc05-2123

[27] Scott LJ, Mohlke KL, Bonnycastle LL, Willer CJ, Li Y, et al (2007) A genome-wide association study of type 2 diabetes in Finns detects multiple susceptibility variants. Science 316: 1341–1345. 17463248 10.1126/science.1142382 PMC3214617

[28] Stancáková A, Javorský M, Luulasmaa T, Haffner SM, Kuusisto L, et al (2009) Changes in insulin sensitivity and insulin release in relation to glycemia and glucose tolerance in 6416 Finnish men. Diabetes 58: 1212–1221. 19223598 10.2337/db08-1607 PMC2671053

[29] Hertel JK, Johansson S, Sonestedt E, Johsson A, Lie RT, et al (2011) FTO, type 2 diabetes, and weight gain throughout adult life: a meta-analysis of 41,504 subjects from the Scandinavian HUNT, MDC, and MPP studies. Diabetes 60: 1637–1644. 21398525 10.2337/db10-1340 PMC3292341

[30] Jacobsen BK, Eggen AE, Mathiesen EB, Wilsgaard T, Njolstad I (2011) Cohort profile: The Tromso Study. Int J Epidemiol DOI doi: 10.1093/ije/dyr049. 10.1093/ije/dyr049 PMC3429870 21422063

[31] Pilia G (2006) Chen WM (2006) Scuteri A (2006) Orrú M (2006) Albai G, et al (2006) Heritability of cardiovascular and personality traits in 6,148 Sardinians. PLoS Genet 2: e132. 16934002 10.1371/journal.pgen.0020132 PMC1557782

[32] Sanna S, Li B, Mulas A, Sidore C, Kang HM, et al (2011) Fine mapping of five loci associated with low-density lipoprotein cholesterol detects variants that double the explained heritability. PloS Genet DOI:10.1371/journal.pgen.1002198. 10.1371/journal.pgen.1002198 PMC3145627 21829380

[33] Bakhtadze E, Cervin C, Lindholm E, Borg H, Nilsson P, et al (2008) Common variants in the TCF7L2 gene help to differentiate autoimmune from non-autoimmune diabetes in young (15–34 years) but not in middle aged (40–59 years) diabetic patients. Diabetologia 51: 2224–2232. 18839133 10.1007/s00125-008-1161-2

[34] Cervin C, Lyssenko V, Bakhtadze E, Lindholm E, Nilsson P, et al (2008) Genetic similarities between latent autoimmune diabetes in adults, type 1 diabetes, and type 2 diabetes. Diabetes 57: 1433–1437. 18310307 10.2337/db07-0299

[35] Lyssenko V, Jonsson A, Almgren P, Pulizzi N, Isomaa B, et al (2008) Clinical risk factors, DNA variants, and the development of type 2 diabetes. N Engl J Med 359: 2220–2232. 19020324 10.1056/NEJMoa0801869

[36] Korn JM, Kuruvilla FG, McCarroll SA, Wysoker A, Nemesh J, et al (2008) Integrated genotype calling and association analysis of SNPs, common copy number polymorphisms and rare CNVs. Nat Genet 40: 1253–1260. 18776909 10.1038/ng.237 PMC2756534

[37] Devlin B, Roeder K (1999) Genomic control for association studies. Biometrics 55: 997–1004. 11315092 10.1111/j.0006-341x.1999.00997.x

[38] Milligan GB (2003) Maximum-likelihood estimation of relatedness. Genetics 163: 1153–1167. 12663552 10.1093/genetics/163.3.1153 PMC1462494

[39] Purcell S, Neale B, Todd-Brown K, Thomas L, Ferreira MA, et al (2007) PLINK: a tool set for whole-genome association and population-based linkage analyses. Am J Hum Genet 81: 559–575. 17701901 10.1086/519795 PMC1950838

[40] Balding DJ, Nichols RA (1995) A method for quantifying differentiation between populations at multi-allelic loci and its implications for investigating identify and paternity. Genetics 96: 3–12. 10.1007/BF01441146 7607457

[41] Kang HM, Sul JH, Service SK, Zaitlen NA, Kong SY, et al (2010) Variance component model to account for sample structure in genome-wide association studies. Nat Genet 42: 348–354. 20208533 10.1038/ng.548 PMC3092069

[42] Scuteri A, Sanna S, Chen WM, Uda M, Albai G, et al (2007) Genome-wide association scan shows genetic variants in the FTO gene are associated with obesity-related traits. PloS Genet 3 doi:10.1371/journal.pgen.0030115. 10.1371/journal.pgen.0030115 PMC1934391 17658951

[43] Howie B, Fuchsberger C, Stephens M, Marchini J, Abecasis GR (2011) Fast and accurate genotype imputation in genome-wide association studies through pre-phasing. Submitted 10.1038/ng.2354 PMC3696580 22820512

[44] Giannoulatou E, Yau C, Colella S, Ragoussis J, Holmes CC (2008) GenoSNP: a variational Bayes within-sample SNP genotyping algorithm that does not require a reference population. Bioinformatics 24: 2209–2214. 18653518 10.1093/bioinformatics/btn386

[45] The International HapMap 3 Consortium (2010) Integrating common and rare genetic variation in diverse human populations. Nature 467: 52–58. 20811451 10.1038/nature09298 PMC3173859

[46] Willer CJ, Sanna S, Jackson AU, Scuteri A, Bonnycastle LL, et al (2008) Newly-identified loci that influence lipid concentrations and risk of coronary artery disease. Nat Genet 40: 161–169. 18193043 10.1038/ng.76 PMC5206900

[47] Loos RJ, Lindgren CM, Li S, Wheeler E, Zhao JH, et al (2008) Common variants near MC4R are associated with fat mass, weight and risk of obesity. Nat Genet 40: 768–75. 18454148 10.1038/ng.140 PMC2669167

[48] Morris AP, Voight BF, Teslovich TM, Ferreira T, Segre AV, et al (2012) Large-scale association analysis provides insights into the genetic architecture and pathophysiology of type 2 diabetes, submitted for publication. 10.1038/ng.2383 PMC3442244 22885922

[49] Cortes A, Brown MA (2011) Promise and pitfalls of the Immunochip. Arthritis Res Ther 13: 101. 21345260 10.1186/ar3204 PMC3157635

