# Correction: The Metabochip, a Custom Genotyping Array for Genetic Studies of Metabolic, Cardiovascular, and Anthropometric Traits

**DOI:** 10.1371/annotation/0b4e9c8b-35c5-4dbd-b95b-0640250fbc87

**Published:** 2013-04-25

**Authors:** Benjamin F. Voight, Hyun Min Kang, Jun Ding, Cameron D. Palmer, Carlo Sidore, Peter S. Chines, Noël P. Burtt, Christian Fuchsberger, Yanming Li, Jeanette Erdmann, Timothy M. Frayling, Iris M. Heid, Anne U. Jackson, Toby Johnson, Tuomas O. Kilpeläinen, Cecilia M. Lindgren, Andrew P. Morris, Inga Prokopenko, Joshua C. Randall, Richa Saxena, Nicole Soranzo, Elizabeth K. Speliotes, Tanya M. Teslovich, Eleanor Wheeler, Jared Maguire, Melissa Parkin, Simon Potter, N. William Rayner, Neil Robertson, Kathleen Stirrups, Wendy Winckler, Serena Sanna, Antonella Mulas, Ramaiah Nagaraja, Francesco Cucca, Inês Barroso, Panos Deloukas, Ruth J. F. Loos, Sekar Kathiresan, Patricia B. Munroe, Christopher Newton-Cheh, Arne Pfeufer, Nilesh J. Samani, Heribert Schunkert, Joel N. Hirschhorn, David Altshuler, Mark I. McCarthy, Gonçalo R. Abecasis, Michael Boehnke

The following information was missing from the Funding section: This works forms part of the translational research themes supported by Barts and The London National Institute for Health Research Cardiovascular Biomedical Research Unit.

