# Supplementary figures and images for: The Metabochip, a Custom Genotyping Array for Genetic Studies of Metabolic, Cardiovascular, and Anthropometric Traits
Source: PLoS Genet. 2012 Aug 2;8(8):e1002793. doi: 10.1371/journal.pgen.1002793 (PMC3410907; doi:10.1371/journal.pgen.1002793)

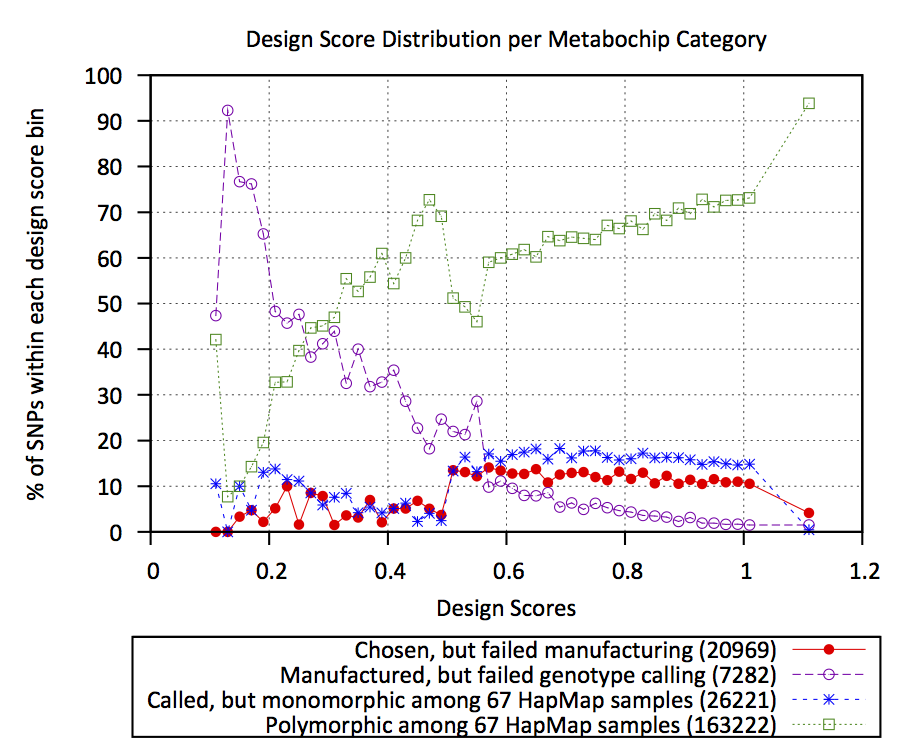

Supplement: Figure S1 — Distribution of Illumina design scores by Metabochip SNP category. (TIFF) [file pgen.1002793.s001.tiff]

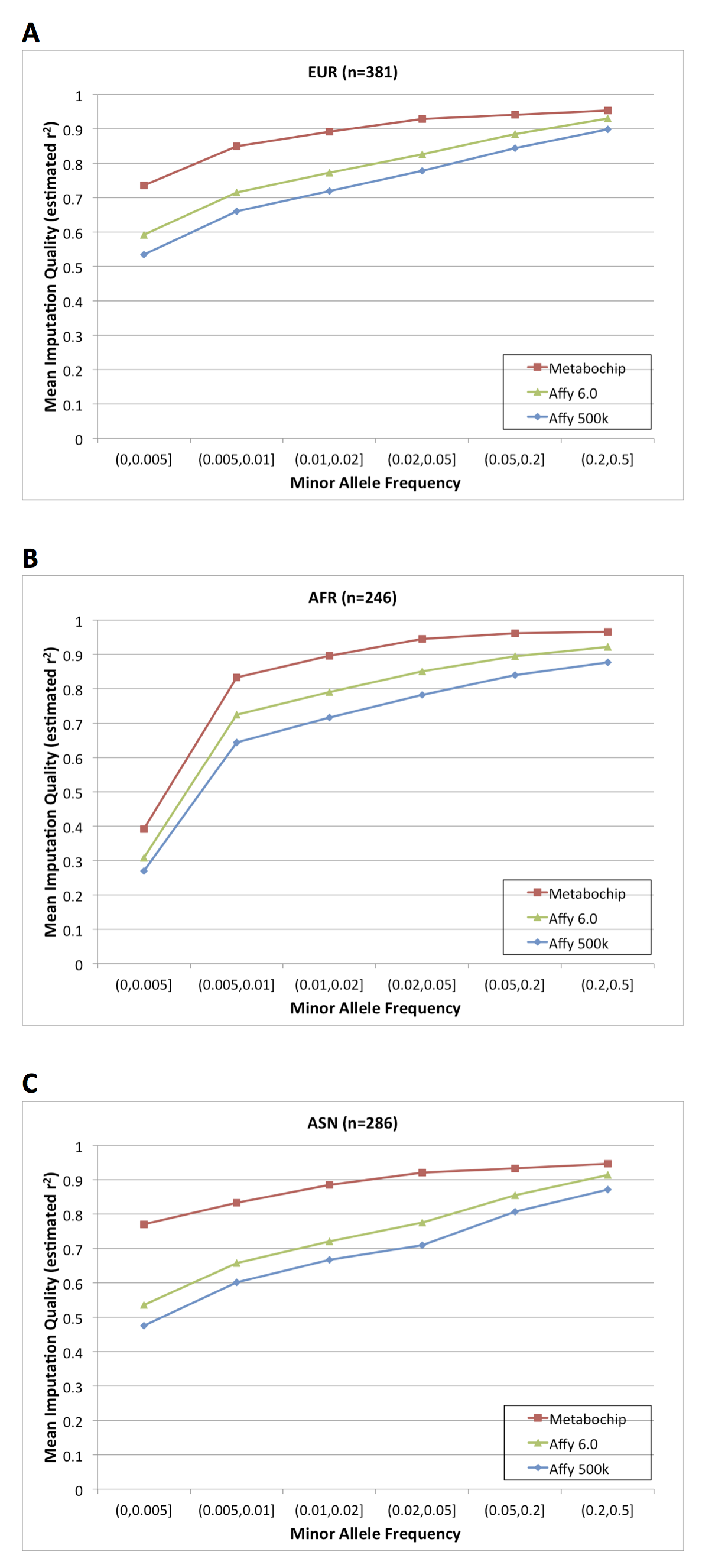

Supplement: Figure S2 — Imputation accuracy in fine mapping regions across three continental populations for (A) Europeans (B) Africans, and (C) East Asians. (TIFF) [file pgen.1002793.s002.tiff]

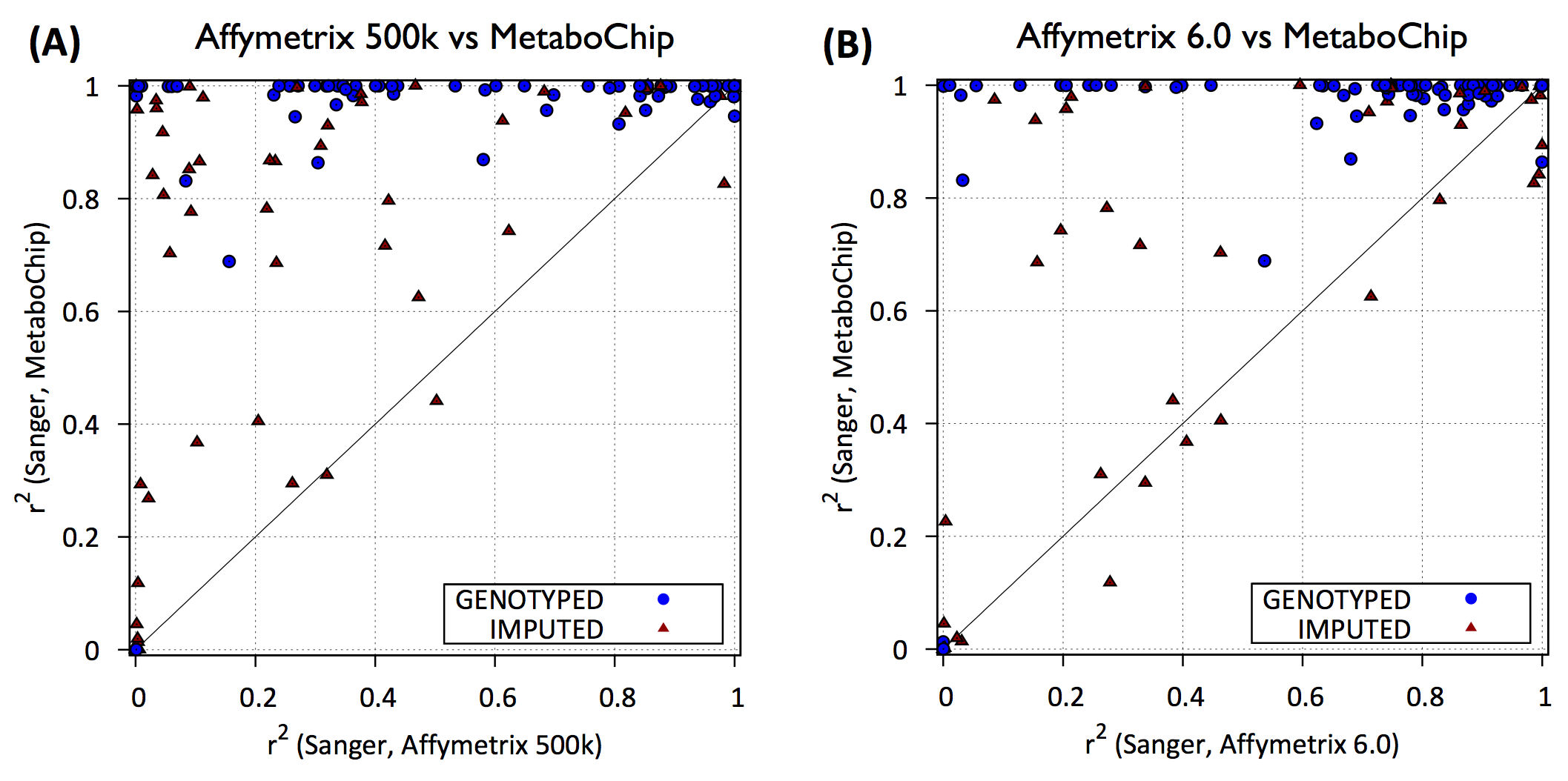

Supplement: Figure S3 — Empirical concordance between Sanger sequencing data and imputed genotypes. Empirical r2 was evaluated between Sanger sequencing data and imputed genotypes from Metabochip or (A) Affymetrix 500 K SNPs and (B) Affymetrix 6.0 SNPs across five loci in 256 Sardinians. (TIFF) [file pgen.1002793.s003.tiff]

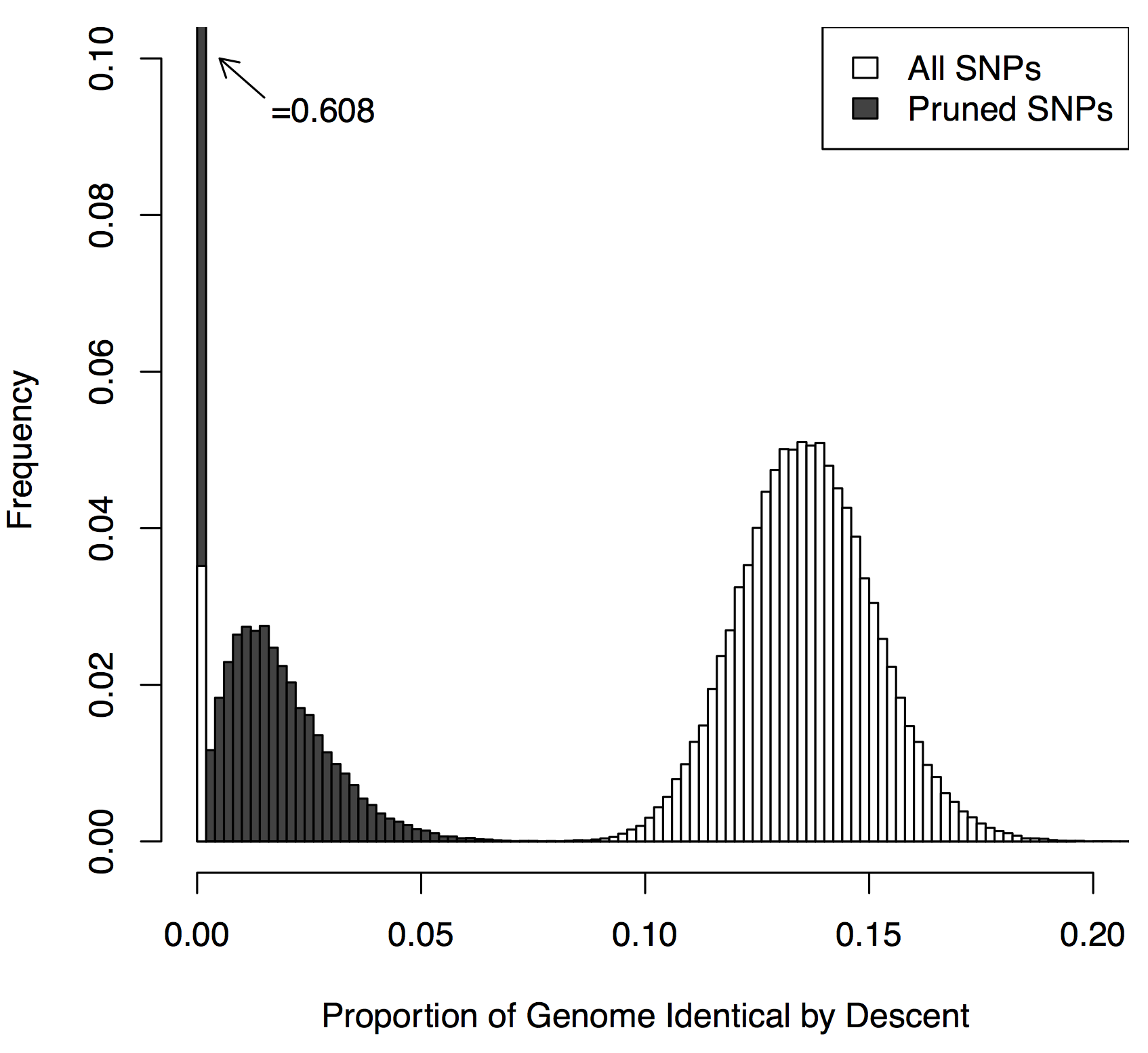

Supplement: Figure S4 — Distribution of estimates of pairwise genome-wide identity-by-descent (IBD) sharing generated by PLINK for all SNPs and for pruned SNPs. (TIFF) [file pgen.1002793.s004.tiff]
