# Supplementary material for: The Metabochip, a Custom Genotyping Array for Genetic Studies of Metabolic, Cardiovascular, and Anthropometric Traits
Source: PLoS Genet. 2012 Aug 2;8(8):e1002793. doi: 10.1371/journal.pgen.1002793 (PMC3410907; doi:10.1371/journal.pgen.1002793)
Supplement: Text S1 — Technical details of SNP selection criteria. (DOC) [file pgen.1002793.s011.doc]

**SUPPLEMENTARY TEXT**

**SNP Selection: Replication**

66,130 unique SNPs (requiring 71,069 array bead types) were chosen for the array based on the union of replication lists provided for each of the eleven Tier 1 (5,000 SNPs for all except for CAD/MI for which 6,516 SNPs were chosen) and twelve Tier 2 (1,000 SNPs) traits. 63,450 SNPs (96%) were successfully designed and manufactured in the array (Supplementary Table S1). Choosing SNPs for the array required (1) linkage disequilibrium (LD)-based pruning of replication lists followed by (2) selection of one or two SNPs from each LD‐grouped set.

*Task One: LD-Based SNP Pruning*

Most consortia provided for each of their traits a list of the 50,000 (Tier 1) or 10,000 (Tier 2) most significant trait-associated (genotyped or imputed) SNPs; for three traits (mean platelet volume, white blood cell count, and platelet count), SNPs with p-value < 10^‐3^ were provided. We requested this 10-fold excess of potential replication SNPs to avoid repeated data requests to the Consortia; in fact, no SNP of rank >42,641 was included for Tier 1 traits. For each trait, listed SNPs were grouped together based on LD r^2^ estimates from the HapMap Phase 2 (HapMap 2) CEU sample. Starting from the most significantly associated SNP and moving to the least significantly associated, a SNP was either grouped with a previous set of SNPs (if r^2^ > 0.2 with the index SNP) or used as an index to seed a new SNP group. All non-HapMap SNPs were included as index SNPs to avoid loss of information.

*Task Two: SNP Selection*

After LD-based grouping, SNP groups were ranked by the p‐value of their index SNP. Within a SNP group, SNPs were ranked by the priority score

PS = 0.2 × R­­P/M + 0.2 × (B - 1) + 0.6 × (1.1 - D)

with lower PS values preferred. Here, RP is the rank of the SNP p-value from 1 (most strongly associated) to M (least strongly associated) within a group, B = 1 or 2 is the number of bead types required for Illumina SNP assay, and D is the Illumina design score of the proposed assay (0 ≤ D ≤ 1 for new SNP assays and D = 1.1 for SNP assays previously manufactured successfully). Using this score, SNPs with the more significant association p‐values, SNPs whose assay required only one bead type, and SNPs with higher assay design scores were favored. Weights 0.2, 0.2, and 0.6 were arbitrary, but were intended to favor SNPs with high design scores. Selected SNPs were spot checked to help ensure sensible choices were made.

Once the SNPs within a group were ordered by priority score, the top SNP for each of the top 5,000 (Tier 1) (6,516 for CAD/MI) or 1,000 (Tier 2) groups was chosen for the array. In addition, the second ranking SNP for the top 300 (Tier 1) or 100 (Tier 2) groups was chosen to guard against failure during the manufacturing process. No minimum Illumina design score filter was applied to this list, although only 850 SNPs with design score < 0.5 were chosen.

**SNP Selection: Fine-Mapping**

139,877 unique SNPs (160,991 bead types) were chosen for the array to fine-map 257 loci nominated by the GWAS meta‐analysis consortia; 122,241 (87%) were successfully designed and manufactured in the array. 214 loci were submitted for signal fine-mapping (SFM) which sought to fine-map the original GWAS signal. 43 loci were submitted for more extensive locus fine-mapping (LFM) which sought to identify all association signals in a consortium-specified region around the index SNP, including rarer causal variants not present in the obvious recombination interval. Consortia defined priority for each locus as high (GOLD), medium (SILVER), or low (BRONZE) based on a number of criteria including strength of association signal, overlap with other traits, and known biology of genes in the region; these priorities were used to determine the degree to which LD-based tagging was used to prune SNP lists within each region (Supplementary Tables S2, S3).

*Task One: Fine-Mapping Boundary Selection*

The 214 SFM intervals were determined using LD estimates from the 1000 Genomes August 2009 release and HapMap 2 CEU data. Initial boundaries were determined by identifying all SNPs satisfying r^2^ ≥ .5 with the index SNP, and then expanding by 0.02 centiMorgans in either direction using the HapMap‐based genetic map. This approach extended the interval in each direction to the nearest flanking recombination hotspot, but stopped if there was no hotspot nearby. Boundaries were then manually screened and adjusted to ensure nearby genes of special interest were included. Boundaries for 28 regions were modified because the interval spanned >500 kb, the r^2^ ≥ .5 boundaries were ambiguous due to a low allele frequency or a single distant SNP in high LD with the index SNP, or based on requests from the relevant consortium representatives. The 43 LFM regions were seeded using similar strategies and then expanded to capture functional units of interest such as nearby coding genes selected by the relevant consortia. Figure 1 demonstrates the difference between a SFM region (yellow) focused on the initial association signal and the more extensive LFM region (blue) which seeks to cover any trait-associated SNP nearby. Before considering overlap, the 257 intervals totaled 64.97 Mb; taking overlap into account reduced this to 45.52 Mb (Table 1).

*Task Two: SNP Selection in Fine-Mapping Intervals*

Within a specified interval, 1000 Genomes SNPs identified by the Sanger Center were considered as candidate fine-mapping SNPs unless SNP minor allele frequency (MAF) was <.01 in all three HapMap 2 samples (CEU, YRI, and combined CHB+JPT). Because individual genotypes and LD estimates for the 1000 Genomes SNPs identified by the Broad Institute were not available at the time of array design, Broad SNPs with MAF <.02 in all three sets were filtered out. SNPs were also filtered out if Illumina design score was <0.5 or if there were any SNPs with CEU MAF ≥ .02 within 15 base pairs. SNPs annotated as nonsynonymous, essential splice site, or stop codons (henceforth "likely functional") were not filtered out based on MAF, design score, or presence of nearby SNPs.

SNPs in LD (r^2^ ≥ .3) with the index SNPs, likely functional SNPs, SNPs specific to the Broad 1000 Genomes calls, and SNPs already included based on other Metabochip design classes were unconditionally chosen for the Metabochip prior to tagging. For SFM GOLD loci, all SNPs that passed filtering criteria were chosen. For LFM GOLD and SFM or LFM SILVER loci, SNPs were tagged at r^2^ = 1.0 across the three HapMap 2 populations, with redundant SNPs added at ~30 kb intervals to ensure coverage of large groups of markers in very strong LD. For SFM or LFM BRONZE loci, we tagged at r^2^ > .8. Our tagging strategy allowed GWAS signals to be well explored and provided SNPs well placed to detect secondary signals within targeted intervals. Numbers of SNPs chosen for each trait and SNP priority class are listed in Supplementary Tables S2 and S3. Tagging resulted in the inclusion of 79.7% of SNPs passing filtering criteria.

**SNP Selection: Prior Trait Associations, CNP Tags, MHC, Wildcards, and Others**

2,210 unique SNPs (2,520 bead types) with design score >0.5 were chosen owing to genome-wide significant association (P < 5 x 10^‐8^) with any trait in http://www.genome.gov/gwastudies/ [1] accessed on August 1, 2009; 2,116 (96%) were successfully manufactured. We augmented this list for each association with one more HapMap 2 CEU SNP (r^2^ > .9 with the index SNP) to guard against genotyping failure and up to four HapMap 2 YRI SNPs (r^2^ > .5) to facilitate investigation of different LD patterns in the YRI sample around the association signal.

6,888 SNPs (7,671 bead types) were chosen based on LD-tagging CNPs from HapMap 2, HapMap 3, or WTCCC+ (Jim Nemesh, Steve McCarroll, Matt Hurles, Richard Pearson, personal communications). All SNPs had design score >0.5; 6,626 (96%) were successfully manufactured.

Three SFM regions were handled differently because they reside in the major histocompatibility complex (MHC) on chromosome 6. Due to high SNP density and limited recombination in the MHC, our standard SNP selection strategy would have included tens of thousands of SNPs per region. Instead, we chose 3,203 SNPs (3,314 bead types) encompassing the 7.5 Mb extended MHC region based on information from the Illumina 660W‐Quad and Cyto SNP‐12 chips [2]; 2,909 SNPs (91%) were successfully manufactured.

144 mtDNA SNPs (147 bead types) were chosen; 135 (94%) were successfully manufactured. These SNPs capture variation down to 5% in Asians and Africans, and to 1% in Europeans [3]; Richa Saxena, personal communication); redundant SNPs were included for SNPs with MAF > .05. Included also were one rare mitochondrial diabetes and deafness (MIDD) mutation (A3243G; [4]) and one putative T2D-associated SNP [5].

90 X and 22 Y chromosome SNPs (112 bead types) were chosen for the array to verify reported sex; 107 (96%) were successfully manufactured. These SNPs had MAF >.1 in all three HapMap 2 samples.

46 SNPs (50 bead types) were chosen for the array and 43 (93%) successfully manufactured to support sample identification (fingerprinting) assays. These markers cover all autosomes and have MAF > .15 in HapMap CEU, CHB, JPT, and YRI samples, and are present on both Illumina and Affymetrix GWAS arrays.

5,323 wild-card SNPs (6,115 bead types) were chosen for the array regardless of Illumina design score; 5,056 (95%) were successfully manufactured. Wild-card SNPs were submitted by GWAS consortia based on consortium‐specific criteria.

In total, 217,695 SNPs (245,241 bead types) were chosen for the array (Table 2). 20,970 SNPs (9.6%) failed during the assay manufacturing process, resulting in 196,725 SNPs available for genotyping. A summary file annotating each Metabochip SNP with ascertainment criteria, SNP assay, and reference strand orientation for alleles is provided at <http://www.sph.umich.edu/csg/kang/MetaboChip/>, and a list of 16 unintentional duplicate SNPs is given in Supplementary Table S4.

**Assessing Overlap Among SNPs Across Traits**

To assess whether the overlap in the SNPs submitted for replication for the different traits showed more or less overlap than expected by chance under the null hypothesis of independence, we carried out a simulation study. As the basis for these simulations, we obtained from each GWAS consortium the list of SNPs they analyzed in their meta-analyses for each of their traits; SNP numbers ranged from ~2.4M to 2.8M. We then extracted genotypes for these SNPs from the founders of the HapMap CEU set (build 23a). For each of at least 1000 simulation replicates per trait pair, we assigned each founder two independent random normal numbers and carried out a GWAS for these random traits using PLINK [6]. The top SNPs were then selected from each trait, with the number chosen to match the actual count of SNPs submitted for that trait (~10,000 or ~50,000). The number of overlapping SNPs in the randomization between the two simulated traits was then counted, and significance assessed by comparing these overlap numbers to those actually observed.

**REFERENCES**

1. Hindorff LA, Sethupathy P, Junkins HA, Ramos EM, Mehta JP, et al. (2009) Potential etiologic and functional implications of genome-wide association loci for human diseases and traits. Proc Natl Acad Sci USA 106:9362-9367.

2. de Bakker PI, McVean G, Sabeti PC, Miretti MM, Green T, et al. (2006) A high-resolution HLA and SNP haplotype map for disease association studies in the extended human MHC. Nat Genet 38:1166-1172.

3. Saxena R, deBakker PI, Singer K, Mootha V, Burtt N, et al. (2006) Comprehensive association testing of common mitochondrial DNA variation in metabolic disease. Am J Hum Genet 79:54-61.

4. Van den Ouweland JM, Lemkes HH, Ruitenbeek W, Sandkuijl LA, de Vijlder MF, et al. (1992) Mutation in mitochondrial tRNA(Leu)(UUR) gene in a large pedigree with maternally transmitted type 2 diabetes mellitus and deafness. Nat Genet 1:368-371.

5. Poulton L, Luan J, Macaulay V, Hennings S, Mitchell J, et al. (2002) Type 2 diabetes is associated with a common mitochondrial variant: evidence from a population-based case-control study. Hum Mol Genet 11:1581-1583.

6. Purcell S, Neale B, Todd-Brown K, Thomas L, Ferreira MA, et al. (2007) PLINK: a tool set for whole-genome association and population-based linkage analyses. Am J Hum Genet 81:559-575.
